# Psychopathic Traits, Externalizing Problems, and Prosocial Behavior: The Role of Social Dominance Orientation

**DOI:** 10.3390/jcm12103521

**Published:** 2023-05-17

**Authors:** Giuseppe Grossi, Francesca Strappini, Enrico Iuliano, Ylenia Passiatore, Francesco Mancini, Valentina Levantini, Gabriele Masi, Annarita Milone, Erica Santaguida, Randall T. Salekin, Pietro Muratori, Carlo Buonanno

**Affiliations:** 1 APC-SPC Scuola di Pscioterapia Cognitiva, 00185 Rome, Italy; 2 Centro di Psicoterapia e Riabiliatzione InMovimento, 04022 Fondi, Italy; 3 Department of Philosophy and Communication Studies, University of Bologna, 40126 Bologna, Italy; 4 Department of Education Sciences, Università Degli Studi Roma Tre, 00154 Rome, Italy; 5 Department of Human Sciences, Università Degli Studi Guglielmo Marconi, 00193 Rome, Italy; 6 IRCCS Stella Maris, Scientific Institute of Child Neurology and Psychiatry, 56018 Pisa, Italy; 7 Institute of Mechanical Intelligence, Scuola Superiore Sant’Anna, 56127 Pisa, Italy; 8 Department of Psychology, The University of Alabama, Tuscaloosa, AL 35487, USA

**Keywords:** social dominance, psychopathic traits, externalizing problems, prosocial behavior, conduct problems

## Abstract

Psychopathic traits in community and referred youths are strongly associated with severe externalizing problems and low prosocial behavior. However, less is known about the mechanisms that may link youth psychopathy and these outcomes. Social dominance orientation (SDO), defined as the general individual orientation toward unequal and dominant/subordinate relationships, might represent a valuable construct to explore to better understand the association between psychopathic traits, externalizing problems, and prosocial behavior. Based on this, the current study aimed to investigate the relationship between psychopathic traits, SDO, externalizing problems, and prosocial behavior in a community sample (*N* = 92, 45.57% females, mean age = 12.53, and *SD* = 0.60) and in a clinical (*N* = 29, 9% female, mean age = 12.57, and *SD* = 0.57) samples of adolescents with Oppositional Defiant Disorder or Conduct Disorder. Results showed that SDO mediated the relationship between psychopathic traits and externalizing problems and between psychopathic traits and prosocial behavior only in the clinical sample. These findings can provide valuable information on psychopathic trait correlates in youths with aggressive behavior disorders; treatment implications are discussed.

## 1. Introduction

Adult psychopathy has been conventionally described as a constellation of traits encompassing interpersonal (deceitfulness and grandiosity), affective (callousness and lack of remorse), behavioral facets (impulsivity and irresponsibility), and antisocial inclination (early conduct problems and delinquent behavior during adolescence) [1,2]. In the attempt to increase our knowledge about the development of severe conduct problems, the construct of psychopathic traits has been extended to childhood and adolescence as well [3,4,5]. Although recent trends in child literature have prevalently focused on a single dimension, the callous-unemotional (CU) traits dimension, child literature has increasingly emphasized the importance of adopting a multidimensional perspective to study youth psychopathy.

As a multifactorial construct, youth psychopathy includes grandiose-manipulative (GM), CU, and daring-impulsive (DI) traits [6]. These dimensions are observable very early in life, are stable from preschool to childhood, up to adulthood [3,4,7,8], and show significant associations with cognitive, affective, and behavioral correlates [4,9,10,11,12].

The current study aimed to investigate the relationship between psychopathic traits, social dominance orientation (SDO), externalizing problems, and prosocial behavior in community and clinical samples of Italian adolescents. We hypothesized that SDO would mediate the relationship between psychopathic traits and externalizing problems, and prosocial behavior. Specifically, we expected that psychopathic traits would be positively associated with SDO [13,14,15], which in turn, would be associated with higher levels of externalizing problems [16,17], but lower levels of prosocial behavior [18,19,20,21].

As already mentioned, there is a renewed interest in investigating youth psychopathy as a multifaceted construct, and this is also demonstrated by the recent development of the Proposed Specifiers for Conduct Disorder (PSCD) scale [6]. The PSCD, which is one of the measures employed in the current study, is a theory-driven measure to assess psychopathic traits in children and adolescents that has now been validated in several studies [10,11,22,23,24,25,26]. It is the only available questionnaire assessing GM, CU, and DI traits in conjunction with conduct disorder (CD) symptoms [6]. The value of including CD symptoms is supported by evidence showing relevant associations between the other dimensions and CD, supporting the need to take into account the combination of CD symptoms with high levels of *all* psychopathic traits—not only CU—to better predict severe outcomes and psychosocial maladjustment in children and adolescents [27,28,29].

Aggressive behavior and externalizing problems are core features of young people with high levels of psychopathic traits in community, clinical, and forensic samples and across different cultures [9,10,11,22,24,25]. Muratori et al. [11] found, in a sample of youths aged 11–14, that the PSCD total score was associated with greater parent- and teacher-reported externalizing problems (i.e., conduct problems and hyperactivity symptoms). Consistently, in a large community sample of Chinese adolescents, Luo et al. [10] found an association between high psychopathic traits, reactive and proactive aggression, and conduct problems. Comparable results were also obtained by Elhami Athar et al. [22] in a sample of Iranian adolescents.

Psychopathic traits have also been related to lower prosocial behavior in children and adolescents. Of note, most of the available findings pertain to a single facet of psychopathy, namely CU traits [30,31]. Milledge et al. [32] found in a sample of referred children and adolescents (aged 5–17 years) that both parent- and teacher-reported CU traits were related to difficulties with prosocial behavior. Even if less is known about the relationship between prosocial behavior and all the other psychopathy dimensions, previous studies have shown that narcissism and impulsivity are significant predictors of prosocial behavior during childhood [33]. Muratori et al. [11] found that higher PSCD total scores were related to less prosocial behavior at home and at school. Moreover, the extant literature suggests that individuals with elevated psychopathic traits or those who are at risk of developing psychopathy present with impairments in a variety of other domains (e.g., eye gaze and orienting to others, laughter, and empathy) that foster and/or are strongly connected to prosocial behavior [34,35].

A possible meaningful construct that might further our understanding of the relationships between youth psychopathy, externalizing/aggressive behavior, and poor prosociality is the social dominance orientation. SDO has been defined as a general individual orientation toward unequal and dominant/subordinate relationships, and captures individuals’ desire for social dominance and superiority for themselves and/or their group [19,36]. It reflects a competitive view of the world and encompasses attitudes and beliefs that some individuals are intrinsically superior (or inferior) to others [37,38].

Some personality traits, including aggressiveness, vindictiveness, and coldness, can predict one’s desire for social dominance [39,40,41], and this kind of drive can widely influence individuals’ interpersonal functioning and behavior [15,37]. In this regard, studies have shown that psychopathy is linked to tendencies toward dominance in community and forensic samples, and that different indices of psychopathy correlate with observer- and self-ratings of dominant behavior [13,14,15]. Overall, these findings suggest that power and dominance are salient cues for individuals with high psychopathic traits and might trigger specific forms of dominant behaviors, such as aggressiveness [15].

Furthermore, studies have shown that SDO was related to different types of aggression and that, more generally, reaching a popular and dominant position among peers, when emphasized as a priority, may lead to aggressive behavior [42,43]. For instance, Mayeux [16] found a positive association between SDO and relational aggression in a sample of adolescents; similarly, Gumpel and Gotdiner [17] found SDO to be associated with both relational and physical aggression.

Finally, SDO has been associated with selfishness, reduced prosocial behavior, a general tendency to care more about one’s own interests than about others’, and reduced willingness to help other people [18,19,20,21]. Within this line of research, Bai et al. [20] found in a sample of students aged 12–18 years that those with more pronounced SDO were more likely to engage in selfish, less prosocial behaviors than those with low SDO. Likewise, a study by Yang et al. [19] demonstrated that SDO was significantly and negatively associated with prosocial behavior in a large sample (*N* = 4246) of Chinese adolescents.

Despite the wide literature confirming an association between psychopathic traits and externalizing problems, and poor prosocial behavior, research is still needed to improve our knowledge about the mechanisms that may link youth psychopathy and these adverse outcomes, as it would increase our understanding of its development, positively impacting case conceptualization as well as preventative and therapeutic strategies.

## 2. Materials and Methods

### 2.1. Participants and Procedure

The community sample included 122 students (mean age = 12.47, *SD* = 0.64) enrolled in a secondary school in Central Italy. Thirty participants had incomplete data (i.e., one of the main measures was not available), so statistical analyses were conducted on a sample of 92 students (45.57% females, mean age = 12.53, *SD* = 0.60).

The clinical sample included 29 adolescents (9% female, mean age = 12.57, *SD* = 0.57) referred from January 2022 to July 2022 to a specialized service for children with behavioral problems. Twenty-five participants received an Oppositional Defiant Disorder (ODD) diagnosis, while the remaining five had a Conduct Disorder diagnosis based on the Schedule for Affective Disorders and Schizophrenia for School-Age Children-Present and Lifetime Version [44].

Participants from the community sample were asked to complete the questionnaires during school hours, while those from the clinical sample completed them at the hospital during their referral. Participants’ parents were also required to complete a questionnaire.

The participants were informed about the purpose of the study, and parents and adolescents signed an informed consent form. The study conformed to the Declaration of Helsinki, and the Regional Ethical Committee (Meyer Hospital, Florence) approved the study (N. 64/2019).

### 2.2. Measures

***Proposed Specifiers for Conduct Disorder.*** The PSCD was used to assess psychopathic traits, namely GM, CU, and DI traits, along with symptoms of CD (Salekin, 2016, 2017) [4,6]. In the current study, we used the Italian parent-report version of the PSCD, validated by Muratori et al. [11]. Items (e.g., “*I can turn on the charm in any situation*,” and “*I have engaged in physical aggression against animals or people*”) are rated on a 3-point Likert scale, with responses ranging from 0 (*not true*) to 2 (*true*). Following Muratori et al. [11], items 3, 12, 14, 16, and 17 were deleted. Previous studies have shown that the PSCD scale has satisfactory internal consistency and is associated with alternative measures of psychopathic traits, conduct problems, aggressive behavior, and externalizing symptoms [9,10,11,22,23,24,45]. Higher scores indicate higher levels of psychopathic traits. In the current study, we used the PSCD total score to represent the broader construct.

***Social Dominance Orientation.*** The participants completed the self-report Italian translation of the social dominance orientation (SDO) scale [37,46]. The SDO scale consists of 13 items measuring individuals’ social dominance attitude. Participants were asked to indicate their agreement with each item (e.g., “*Certain groups of people are simply not the same as others*,” and “*To get ahead in life, it’s sometimes necessary to step on other groups*”) on a 5-point Likert scale, ranging from 0 (*totally disagree*) to 4 (*totally agree*). Higher scores indicated higher levels of SDO.

***Strengths and Difficulties Questionnaires.*** Participants’ externalizing problems and prosocial behavior were assessed with the Italian parent-report version of the Strengths and Difficulties Questionnaire (SDQ) [47,48,49]. The SDQ assesses emotional symptoms, peer problems, hyperactivity inattention, conduct problems, and prosocial behavior. Each subscale includes 5 items, rated on a 3-point Likert scale (0 = *not true*, 1 = *somewhat true*, and 2 = *certainly true*). Its score ranges from 0 to 10, with higher scores suggesting greater difficulties, except for prosocial behavior. Based on Goodman et al. [50] findings, we combined the conduct and hyperactivity symptoms subscales into an externalizing problems score.

### 2.3. Statistical Analyses

Descriptive statistics were computed for each measure and each sample. The questionnaires’ internal consistency was determined by mean interitem correlations (MICs). The optimal range of MIC is 0.15–0.50; MICs below 0.15 indicate that items are not well correlated and likely not measuring the same construct in a proper way, while MICs above 0.50 suggest that items might be repetitive and redundant. Multiple Pearson correlation coefficients were estimated to examine the relationship between the study variables.

Mediation analyses were performed to examine whether SDO mediated the relationship between the PSCD total score and externalizing problems or prosocial behavior separately in the community and clinical samples. The direct effect of the independent variable (PSCD) on the dependent variable (SDQ) and the indirect effect through the SDO on SDQ were estimated. Mediation analyses were performed using the medmod package in Jamovi 2, and the level of statistical significance was set at *α* = 0.05. Bootstrapping was performed [51], and the values of 10,000 bootstrap samples were selected and considered sufficient for the mediation [52].

## 3. Results

Descriptive statistics showed that participants in the clinical group had higher scores in all the measures except for the prosocial behavior subscale of the SDQ, where the score was significantly lower (Table 1). As regards the PSCD scores, we found that 10.70% of the children in the clinical sample and 2.2% of those in the community sample reported high psychopathy features (PSCD mean total score > 1; see [10]).

Internal reliability was calculated for each scale. MIC for the SDO was 0.32 in the clinical sample and 0.22 in the community sample; for SDQ externalizing problems, it was 0.27 in the clinical sample and 0.23 in the community sample; for prosocial behavior, it was 0.29 in the clinical sample and 0.22 in the community sample. As regards the PSCD total score, MIC was 0.19 for the clinical sample and 0.21 for the community sample.

Bivariate correlations (Table 2) showed that, in the clinical sample, the SDO score was positively and significantly associated with externalizing problems (*r* = 0.765, *p* < 0.001, and 95% CI: [0.55 0.89]) and negatively associated with prosocial behavior (*r* = −0.677, *p* < 0.001, and 95% CI: [−0.84 −0.41]). Similarly, in the community sample, the SDO score was positively and significantly associated with externalizing problems (*r* = 0.216, *p* = 0.039, and 95% CI: [0.01 0.40]) and negatively associated with prosocial behavior (*r* = −0.239, *p* = 0.022, and 95% CI: [−0.42 −0.04]). As regards the associations between the PSCD and SDO total scores, they were positively and significantly associated in the clinical sample (*r* = 0.752, *p* < 0.00, 195%, and CI: [0.53 0.88]) but not in the community one (*r* = 0.172, *p* = 0.102, and 95% CI: [−0.035 0.365]).

**Figure 1:**
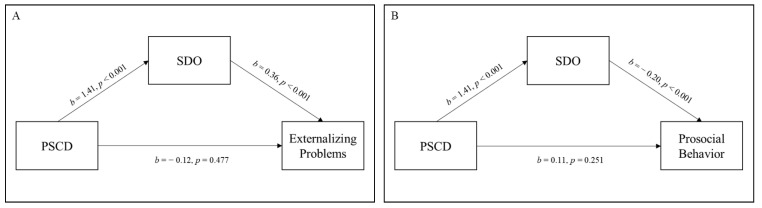
Relationships between Proposed Specifiers for Conduct Disorder score, social dominance orientation and externalizing problems (**A**), and prosocial behavior (**B**) in the clinical sample. *Note*. PSCD = Proposed Specifiers for Conduct Disorder; SDO = social dominance orientation.

Mediation analyses indicated that SDO mediated the relationship between PSCD and externalizing problems in the clinical sample (Figure 1A). Specifically, findings showed a significant indirect effect of psychopathic traits on externalizing problems via SDO (*b* = 0.51, 95% CI: [0.25 0.93], and *p* = 0.003; indirect effect accounting for 81.1% of the total effect), with PSCD total score positively associated with SDO (*b* = 1.41, 95% CI: [0.99 1.97], and *p* < 0.001), which in turn was positively associated with externalizing problems (*b* = 0.36, 95% CI: [0.22 0.52], and *p* < 0.001). The direct effect of PSCD on externalizing problems was not significant (*b* = −0.12, 95% CI: [−0.52 0.14], and *p* = 0.477; direct effect accounting for 18.9% of the total effect). Furthermore, SDO mediated the relationship between PSCD and prosocial behavior in the clinical sample (*b* = −0.29, 95% CI: [−0.53 −0.12], and *p* = 0.005; indirect effect accounting for 71.9% of the total effect) (Figure 1B). Specifically, the PSCD total score was positively associated with SDO (*b* = 1.41, 95% CI: [0.97 1.95], and *p* < 0.001), which in turn, was negatively associated with prosocial behavior (*b* = −0.20, 95% CI: [−0.32 −0.10], and *p* < 0.001). The direct effect of psychopathic traits on prosocial behavior was not significant (*b* = 0.112, 95% CI: [−0.05 0.33], and *p* = 0.251; direct effect accounting for 28.1% of the total effect).

**Figure 2:**
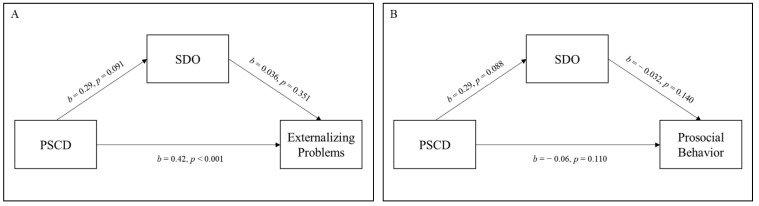
Relationships between Proposed Specifiers for Conduct Disorder score, social dominance orientation and externalizing problems (**A**), and prosocial behavior (**B**) in the community sample. *Note*. PSCD = Proposed Specifiers for Conduct Disorder; SDO = social dominance orientation.

As regards the community sample, we found a direct effect (*b* = 0.42, 95% CI: [0.26 0.58], and *p* < 0.001; direct effect accounting for 97.93% of the total effect) but not an indirect effect of PSCD total score on externalizing problems (*b* = 0.01, 95% CI: [−0.01 0.04], and *p* = 0.489; indirect effect accounting for 2.07% of the total effect) (Figure 2A). Finally, in the community sample, psychopathic traits were not directly or indirectly (*b* = −0.01, 95% CI: [−0.03 0.01], and *p* = 0.321 indirect effect accounting for 11.3% of the total effect) associated with prosocial behavior.

## 4. Discussion

Psychopathic traits in community and referred youths are associated with more severe and persistent externalizing problems [9,10,11,22,24,25] and lower prosocial behavior [11,32,33]. In order to improve our understanding of these burdensome problems and the effectiveness of our interventions, exploring the link between psychopathic traits and negative outcomes may be crucial. Investigating the mechanisms underlying these associations could help identify possible risk factors associated with specific adverse outcomes and new intervention targets. This is particularly relevant since youths with psychopathic traits are usually less responsive to traditional interventions, and more effective and tailored preventive and treatment models are currently considered a priority by clinicians and researchers. Accordingly, the current study sought to explore the relationship between psychopathic traits, social dominance, externalizing problems, and prosocial behavior in community and clinical samples of Italian adolescents. Specifically, we tested the hypothesis that social dominance mediated the link between psychopathic traits and parent-reported externalizing problems and prosocial behavior.

Correlations revealed that, in the community sample, higher scores of the PSCD were associated with higher externalizing problems, which was consistent with the available literature, e.g., [10,11]. In line with previous studies [16,17,19,20], we also found an association between SDO and externalizing problems and poor prosocial behavior. This suggests that a greater tendency to believe that some individuals are intrinsically superior to others and a broad desire for social dominance might contribute to youths externalizing problems and reducing prosocial and cooperative behavior.

In contrast with our hypotheses, in the community sample, the social dominance orientation did not mediate the relationship between psychopathic traits and externalizing problems or prosocial behavior. We only found evidence of a direct effect of psychopathic traits on externalizing problems, with the PSCD total score positively associated with externalizing problems. Along with correlations, this evidence further emphasizes the importance of psychopathic traits as a risk for externalizing problems, even in the general population. However, once modeled in the mediation models, psychopathic traits were unrelated to social dominance, and dominance tendencies were not associated with externalizing problems or prosocial behavior.

In the clinical sample, we found that higher psychopathic traits were correlated with higher levels of externalizing problems, lower prosocial behavior, and more pronounced SDO. These results are consistent with studies showing the association between psychopathic traits, social dominance and externalizing problems, and prosociality, e.g., [27,39,41].

As regards the mediation models, consistent with our hypotheses, we found that SDO mediated the relationship between the PSCD psychopathic traits and externalizing problems, and prosocial behavior. Specifically, higher PSCD scores were related to a greater dominance orientation, which, in turn, was associated with higher levels of parent-reported externalizing problems and lower prosocial behavior.

Previous research has shown that in adults, psychopathy is generally associated with self-enhancing motivations, including having a significant social position and gaining power, and a lower interest in self-transcendent goals, which emphasizes concern for the well-being of others [53,54]. Being popular and powerful are so-called extrinsic goals, which revolve around obtaining rewards and/or praise [55]. Individuals driven by these goals are more prone to competition than cooperation [56]. It is plausible, then, to suggest that the behavior of psychopathic individuals (i.e., high externalizing problems, low prosocial behavior) might be partially caused by being prone to obtaining power and high social positions (i.e., high SDO) as opposed to community and prosocial efforts [53], as shown by our findings. Aggressive and externalizing behaviors may be seen as useful and proper ways to gain and maintain social dominance by adolescents with psychopathic traits. This is also consistent with findings demonstrating that aggressive and externalizing behaviors in youths with high psychopathic traits are frequently driven by the desire to dominate others and pursue their own goals, and are considered appropriate tools to manage social relationships and conflicts [31,57]. Moreover, as some previous studies have shown, aggressive behaviors can serve this aim [58,59], increasing the chance that youths with elevated psychopathic traits may more consistently use these strategies to deal with others. Finally, social dominance may represent the belief that the world is a “ruthlessly competitive jungle in which might is right, the strong win, and the weak lose” [36], which may imply a lack of concern about harming others and a lack of concern regarding fairness. Such perspectives are typically found in individuals with elevated psychopathic traits [60]. This vision of society might lead psychopathic youths to objectify others, take advantage of them, and move forward in life in a manipulative and callous manner [61], which likely results in a low level of prosociality and altruism.

Various factors might account for the different results from the mediation analysis in the two samples. First, only 2.2%—which is a rather low percentage compared to evidence from previous studies [10]—of the participants in our community sample reported high levels of psychopathic traits. Additionally, participants from the community sample reported significantly lower SDO and PSCD scores than the clinical ones [17,19,20], and, as shown by the zero-order correlations, the two constructs were not significantly correlated in the community sample. It is possible to advance that social dominance may be more subtle in community samples and, therefore, not as easily detected. Additionally, social dominance and its related beliefs might have a more significant impact on the clinical sample, which consisted of youths with ODD and CD. Thus social dominance and its related beliefs may vary in their specific influence and may have an impact on ODD/CD adolescents’ behavior and interpersonal functioning due to their specific cognitive and socio-emotional features (e.g., emotion processing deficits, hostile attribution bias, poor verbal abilities, high moral disengagement) [62,63,64,65,66].

The findings of the current study need to be considered in light of some limitations. First, the small sample sizes might have reduced the power of our statistical analyses and influenced the mediation analysis results. Indeed, it is easier in small samples to find evidence of complete mediation, which might explain the absence of a direct effect of psychopathic traits on externalizing problems and prosocial behavior in the clinical sample. However, recent studies using the PSCD in community and clinical samples were conducted with similar sample sizes [26]. Moreover, future research should explore the role of other variables that might be related to the topic of our study. For instance, SDO reflects a sense of superiority, so it would be interesting to investigate whether some parenting dimensions and parents’ characteristics typically associated with narcissistic self-views (i.e., parents’ narcissistic traits and parental overvaluation) [67] might also be implicated in the relationships identified in the current study.

Despite these limitations, our study has relevant implications. First, our results provide new evidence related to the utility of the PSCD as a tool to assess youths’ psychopathic traits. Specifically, this is the first study to use the PSCD in a sample of youths with ODD and DC and explore the association between the PSCD score with not only behavioral measures but also SDO, a cognitive variable that is poorly explored in young populations. Additionally, our results, in line with several previous studies, confirmed a significant association between psychopathic traits and externalizing problems, further emphasizing the importance of implementing preventive interventions (e.g., school-based prevention programs) to reduce the psychopathological risk of youths with high levels of psychopathic traits. The results of this study also call for relevant considerations for the treatment of adolescents with behavioral problems and psychopathic traits. Dominance orientation and motivation can influence treatment alliance and the willingness to follow therapists’ directions and advice, with negative implications for the outcomes as well [15]. Consistently, previous studies have shown that dominant tendencies in patients are associated with poorer post-treatment and follow-up improvements [68]. This aspect might be of particular relevance, especially for adolescents with high levels of psychopathic traits. Indeed, due to their developmental stage, adolescents may be more resistant to therapy and challenging to engage in therapeutic processes [69]. Moreover, psychopathic traits are among the most significant features that hamper treatment alliance in adults and youths. As such, individuals with psychopathic traits are usually considered a challenge to treat [70,71,72].

The current study demonstrated that, in the clinical sample, social dominance mediated the relationship between psychopathic traits and greater externalizing problems, and lower prosocial behavior. This evidence suggests, consistent with previous studies [15], that dominance orientation/motivation should represent a target of treatment interventions. Indeed, reshaping youths’ view of the world as an unhealthy competitive environment in which some individuals are superior to others and, therefore, should dominate others might influence their interpersonal behavior. Therefore, reducing the feeling of superiority and the need to dominate others may reduce the emission of aggressive and hostile behavior in dealing with other people and allow for more cooperative efforts to obtain goals. Taken from a different perspective, and as emphasized by Johnson et al. [15], “heightened dominance motivation could be channeled in a positive way that supports interpersonal connectedness.” Different strategies may be used to establish cooperative behaviors and reduce aggression toward others. Efforts toward social dominance usually come with a cost and have negative long-term consequences. Instead, prosocial and altruistic behaviors can serve this same goal while also leading to strong bonds and friendships with others, fostering positive developmental outcomes [59,73]. In order to promote this shift from coercive to more affiliative strategies to rise in rank, therapies should address youths’ social and self-regulation skills, which are the underpinnings of cooperation, prosociality, and helping behaviors.

## Figures and Tables

**Table 1:** Descriptive statistics.

	Community Sample (*N* = 92)		Clinical Sample (*N* = 29)				
	*Mean*	*SD*	*Mean*	*SD*	*t*	*p*	*d*
PSCD total score	8.58	4.42	14.00	4.04	−5.97	<0.001	−1.25
SDO	19.68	6.60	22.96	7.58	−2.22	0.028	−0.48
SDQ—Externalizing	3.57	3.13	7.93	3.10	−6.30	<0.001	−1.36
SDQ—Prosocial	8.23	1.64	6.39	1.77	5.09	<0.001	1.10

*Note*. PSCD = Proposed Specifiers for Conduct Disorder; SDO = social dominance orientation; SDQ = Strengths and Difficulties Questionnaire; and SD = Standard Deviation.

**Table 2:** Zero-order correlations among the study variables in the community (above the diagonal) and clinical (below the diagonal) samples.

	SDO	PSCD	Externalizing Problems	Prosocial Behavior
SDO	-	0.172	0.216 *	−0.239 *
PSCD	0.752 ***	-	0.609 ***	−0.200
Externalizing Problems	0.765 ***	0.509 **	-	−0.332 **
Prosocial Behavior	−0.677 ***	−0.399 *	−0.750 ***	-

*Note*. SDO: social dominance orientation; PSCD: Proposed Specifiers for Conduct Disorder. * *p* < 0.05, ** *p* < 0.01, and *** *p* < 0.001.

## Data Availability

Data are available from the corresponding author upon reasonable request.
